# Ten simple rules for designing learning experiences that involve enhancing computational biology Wikipedia articles

**DOI:** 10.1371/journal.pcbi.1007868

**Published:** 2020-05-14

**Authors:** Alastair M. Kilpatrick, Audra Anjum, Lonnie Welch

**Affiliations:** 1 MRC Centre for Regenerative Medicine, University of Edinburgh, Edinburgh BioQuarter, Edinburgh, United Kingdom; 2 Office of Instructional Innovation, Ohio University, Athens, Ohio, United States of America; 3 School of Computer Science and Electrical Engineering, Ohio University, Athens, Ohio, United States of America; Carnegie Mellon University, UNITED STATES

## Introduction

Wikipedia is the largest and most visited encyclopedia on the World Wide Web [1]. Wikipedia is frequently accessed as an educational resource in computational biology, with the articles on Bioinformatics and CRISPR being viewed 413,000 and 1.18 million times, respectively, in 2019 [2]. However, academics remain skeptical of Wikipedia as a reliable source of knowledge [3]. A common complaint by educators is the perceived lack of quality of information found on Wikipedia. Some educators also worry that the platform discourages deeper learner engagement, providing learners with a crutch instead of ways to engage in rigorous, secondary research within a discipline. Both of these concerns often overshadow the advantages of free and readily available knowledge. Given that Wikipedia is one of the most visited websites and an established platform for knowledge seekers, it makes sense to address these concerns and help learners make the most of what they would do anyway.

Mentored contributions from students to open platforms like Wikipedia offer opportunities for improved rigor, quality, depth, and reliability of the information indexed and make it relatable to a wide audience. There have been stellar examples of such mentored contributions, resulting in well-curated additions to domain-specific knowledge amenable for consumption by both public and specialist audiences. For instance, educators around the world have mentored students to either improve or create Wikipedia articles and enter the annual International Society for Computational Biology (ISCB) Wikipedia Competition, a grassroots initiative designed to improve the coverage and depth of computational biology topics [4,5]. Entries are judged based on the quality of writing, figures, and depth of subject knowledge [6]. Winning entries have included important topics in computational biology, such as chromosome conformation capture [7], molecular phylogenetics [8], and the Ruzzo-Tompa algorithm [9].

Some educators have gone further, replacing the writing of traditional term papers with the rigorous editing or creation of new articles in Wikipedia, resulting in class assignments that enhance a vital, publicly accessible resource of field-specific information. Trainees at 24 United States universities participated in a pilot of the Wikipedia Education Program [10] during the 2010–2011 academic year. A 2012 article reviewed the experiences of four professors who participated, each assigning trainees to write Wikipedia articles on a course topic in place of a term paper [11]. Although each professor tailored assignments to her/his particular class, all found the assignments “extremely useful” in improving trainees’ learning. Similar positive outcomes have been reported in other studies, including larger introductory courses of over 100 learners [12]. While recognizing that there may be an element of confirmation bias in these articles [3], there is an emerging consensus that is highly supportive of editing Wikipedia articles as part of a class curriculum. The scope of this work does not have to be limited to the traditional classroom. Using Wikipedia lends itself to a variety of learning and teaching contexts throughout the professional development path, including single-semester bioinformatics courses, short and intensive bioinformatics courses, and professional community service activities.

Although there are advantages to facilitating mentored student contributions to Wikipedia, bioinformatics educators face two main challenges in designing meaningful learning experiences: first, ensuring the quality of students’ contributions to Wikipedia, and second, inculcating meaningful learning activities that align with intended outcomes. In addressing the first challenge, we highlight substantial evidence that shows that writing can be an effective tool to promote learning in the classroom [13]. A “writing-to-learn” pedagogical approach focuses on deepening understanding and improving long-term retention of content and concepts through writing activities [14,15]. The inclusion of varied writing activities in a computer science course compels students to be analytical and critical in their thinking, resulting in improved long-term retention of course content and greater awareness of different writing styles [16]. Therefore, integrating well-planned learning experiences and assignments centered around writing is key to ensuring the quality of Wikipedia contributions. It is especially important for science educators, as written communication is often underemphasized in scientific courses.

In addressing the second challenge, we propose that authentic learning may hold the key. Educational researchers have emphasized the value of authentic learning as a part of learner-centered teaching [17]. Authentic learning elements are already commonplace in computational biology curricula through the use of authentic databases, algorithms, and research tools [18,19], and a Wikipedia-based writing project is a natural extension. Themes of authentic learning include (a) using real-world problems to engage learners in professional work, (b) providing inquiry activities that practice thinking skills and metacognition, (c) encouraging discourse among communities of learners, and (d) empowering learners through choices [20]. A Wikipedia-based writing activity offers a more authentic learning experience than a traditional term paper [21,22] and provides trainees with the opportunity to practice disseminating domain-specific knowledge to a broad audience while navigating the complexity and ambiguity of working through a real-life problem. Importantly, these learning outcomes overlap with core competencies from the ISCB’s bioinformatics training and education framework [23,24].

The benefits of authentic learning experiences are well documented; however, preparing for authentic learning may present logistical and instructional challenges [17,25]. Therefore, we present Ten Simple Rules for bioinformatics educators who wish to use the enhancement of Wikipedia articles as a class project. These rules extend previously published rules for editing Wikipedia more generally [26]; we recommend following these previous rules, which remain an excellent foundation for Wikipedia editing.

## Rule 1: Use Wikipedia to foster ISCB core competencies

Since 2014, the Curriculum Task Force of the ISCB Education Community of Special Interest (COSI) has sought to define, apply, evaluate, and refine core competencies: proficiencies that are desirable for learners to attain in order to succeed in a breadth of careers in the field of bioinformatics [23,24,27]. A relevant subset of the ISCB core competencies is presented in **Table 1**. You should consider how your courses currently provide training in these competencies and how these competencies could be fostered by incorporating Wikipedia-based elements.

**Table 1:** Selected ISCB bioinformatics core competencies.

Label	Competency
I	GUI/web-based computing skills appropriate to the discipline (e.g., effectively use bioinformatics and analysis tools through the web).
L	Local and global impact of bioinformatics and genomics on individuals, organizations, and society.
M	Professional, ethical, legal, security, and social issues, and responsibilities of bioinformatics and genomic data in the workplace.
N	Effective communication of bioinformatics and genomics problems/issues/topics with a range of audiences, including but not limited to other bioinformatics professionals.
O	Effective teamwork to accomplish a common scientific goal.
P	Engage in continuing professional development in bioinformatics.

This table provides a subset of the current ISCB bioinformatics core competencies relevant to enhancing computational biology Wikipedia articles as a class project (the competency labels correspond to those used by Mulder and colleagues [24]).

Abbreviations: GUI, graphical user interface; ISCB, International Society for Computational Biology.

The ISCB core competencies were first defined by surveying bioinformatics core facility directors, career opportunities, and existing bioinformatics curricula [27]. In their latest iteration [24], these competencies are divided into three categories: biological, computational, and professional. Without being prescriptive, the ISCB core competencies provide a framework in which best practices in bioinformatics education can be identified.

The extent to which domain-specific (i.e., biological and computational) core competencies are achieved by learners depends on the scope of your course or training sequence. Incorporating a Wikipedia-based writing element affords learners the opportunity to develop core competencies that emphasize professional skills while enhancing both domain knowledge and writing abilities. These core competencies have transferable elements that will be beneficial in a trainee’s career, regardless of her eventual chosen field.

Each of these competencies is further discussed below. However, here we highlight competency N, a competency that is often neglected in the bioinformatics classroom. Leveraging the worldwide reach of Wikipedia as a platform for trainees’ writing will be beneficial in promoting effective communication of bioinformatics topics to an audience of varying expertise.

## Rule 2: Draw inspiration from the experiences of other educators

Several academic reviews of class projects involving editing Wikipedia articles are available for inspiration and guidance. In addition to the Wikipedia Educational Program, other reviews include that of a month-long undergraduate class project in chronobiology at Washington University in St. Louis [28] and a graduate seminar in plant–animal interactions at the University of Florida [29]. The latter article presents a flowchart illustrating a potential process for editing Wikipedia articles as a semester-long class project. These reviews provide useful guidance for educators planning Wikipedia-based writing tasks. For further inspiration, here we briefly detail the experience of one of the authors. In place of writing a final term paper, students in an upper-level, one-semester Bioinformatics Tools course at Ohio University were asked to enhance a Wikipedia article relating to computational biology. The project began with an introduction to the Wikipedia writing genre (see **Rule 7**). Subsequently, students were required to make three iterative revisions to their chosen article, receiving feedback from both their coach and their peers after each revision. Peer feedback considered the clarity of writing, the perceived depth of knowledge of the subject area, and the quality of additional media (e.g., figures) used to enhance the article. At the end of the project, students were encouraged to submit their articles to the ISCB Wikipedia Competition.

In addition to the overall goal of learning about a bioinformatics-related topic, students learned how to critique articles, enhanced their writing and editing skills, improved their awareness of available resources, and contributed to the international bioinformatics community. Feedback from the students at the end of the semester was positive overall. One student who edited an article relating to a bioinformatics algorithm reported that, “[I] felt that many subtle details of the problems relat[ing to] my topic only really came to my attention when I attempted to write [them] down in words. It was after I finished editing the article that I truly understood every bit of the [algorithm].” Another commented that writing for Wikipedia’s nonexpert audience “encouraged me to develop a broader knowledge of the subject, and to think about how I would explain it clearly to other people.” Two articles developed in the class won awards in the 2016–2017 ISCB Wikipedia Competition [30], with enhancements to the Smith-Waterman algorithm article [31], which included a novel animated illustration of how the algorithm is applied to example data, awarded first prize.

## Rule 3: Design a learning experience that aligns with your curriculum

Maximizing the potential of using Wikipedia requires careful, intentional planning. Designing experiences for learners is best done when attention is paid to alignment with overall teaching goals. Backward design approaches to curriculum development (i.e., beginning with the end goal in mind) ensure that your curriculum design decisions match your intended outcomes [32]. We caution against trying to fit a Wikipedia-based writing assignment into your existing curriculum without considering whether there is enough time for each component of the project to be done well.

A helpful exercise in thinking about how trainees may benefit from a Wikipedia-based assignment is to picture a student standing in front of you at the end of your course. This student received the best possible grade. What can the student do now, as a result of completing your course? What new skills can this student demonstrate? How did a Wikipedia-based project contribute to this change in the student?

The answers to these questions will form your goals, or learning objectives, for the experience. Working backward from there, you can determine what materials, practice, and feedback learners will need to reach these goals. For example, if your goal for the assignment is that learners will be able to describe, infer, compare, or synthesize genomics concepts, then as part of your approach to helping learners reach this goal, you may wish to have learners edit and contribute to Wikipedia articles in genomics, with a particular emphasis on articles that compare genomic concepts.

If Wikipedia editing is to form a significant portion of your course assessment, it will be wise to plan a multiphase, scaffolded learning process, which builds from article review and minor edits, towards independently editing or creating an article. **Rules 4–8** provide guidelines for such a multiphase process.

## Rule 4: Select specific articles

The choice of Wikipedia articles to be edited is an important decision that depends greatly on your course outcomes. If your desired outcomes are more writing focused, then it will make sense to choose less fully developed articles that would benefit greatly from editing. In this case, reviewing and assessing the quality of existing articles could be a useful collaborative classroom activity. Alternatively, if the course is focused on a narrower topic within computational biology, it makes sense to choose articles that fall within this topic.

Article selection may be guided by Wikipedia’s Release Version Tool, which ranks articles relating to computational biology by quality and importance [33]. Article quality is graded according to Wikipedia’s content assessment scale and judged by Wikipedia editors [34]. Computational biology articles may also be ranked by popularity, based on recent page views [35]. You may prefer to curate a focused set of articles for learners to choose from rather than allowing free choice from all computational biology articles.

However, allowing learners to choose their own articles may encourage greater participation than if they are assigned an article. The Wikipedia-based assignment itself meets characteristics “b,” “c,” and “d” of authentic learning activities [36] because it responds to real-life contexts that do not have single or unique solutions, is directed toward a real audience, and provides new information. Alignment with characteristic “a”—the personal frame of reference—can also be satisfied when learners are allowed to choose their articles, define the problem, and select a solution path [20]. For example, a student in the Bioinformatics Tools course described in **Rule 2** chose to enhance a biographical article about bioinformatics pioneer Margaret Oakley Dayhoff. The human-interest element of this project allowed the learner to connect with bioinformatics technology and with the Wikipedia editing project in a very personal way, leading to a significant volume of high-quality edits to the article. In particular, an outstanding request for additional citations dating from February 2013 was resolved and relevant images were added to the article [37].

One additional consideration may be the languages spoken by your trainees, because improving articles in multiple languages will maximize public accessibility of their chosen topics. For instance, the enhancements made to the Smith-Waterman algorithm article mentioned in Rule 2 were simultaneously made to the corresponding article in the English and Chinese Wikipedias, and previous ISCB Wikipedia Competition winners have made contributions to the Spanish Wikipedia. Beyond computational biology, the Wikipedia Education Program has also published case studies for translation assignments [38]. Although it is difficult to assess the veracity or quality of work done in a language you do not know, contributions made in tandem to English and non-English articles (or in any pair of languages) encourage discourse on computational biology topics among those who do not speak English, which is considered to be the scientific lingua franca [39].

## Rule 5: Set clear assessment expectations

In keeping with Wikipedia’s philosophy of openness and its guidelines regarding article quality, we recommend designing and distributing a rubric specific to assessing the Wikipedia contributions of learners. Just as the editing or creating of a Wikipedia article is different from a traditional writing assignment, so the assessment will also be different [40]. Providing your learners with an assessment rubric will help them to understand your expectations for the assignment and will allow them to self-evaluate and reflect on their performance. For educators, having such a rubric also removes a significant amount of guesswork from assessment.

In addition to the aforementioned guidelines for assessing article quality on Wikipedia, the Wiki Education Foundation (which promotes the integration of Wikipedia into coursework by educators in Canada and the US) provides a sample assessment rubric [41], which defines characteristics of assignments from an educational perspective, from “poor” through “excellent.” You will likely want to tailor this sample rubric to your particular assignment; however, the rubric should still reinforce, rather than contradict, Wikipedia’s own rules and style [42]. It is important to emphasize that the unique features of this textual genre offer learners the opportunity to focus on the quality of their message rather than meeting an arbitrary word count. ISCB core competency N, relating to the effective communication of bioinformatics and genomics topics with a range of audiences, is particularly relevant here (see **Table 1**).

If the rubric has been consulted throughout the assignment, then there should be few surprises when it comes to the final grading. A vital difference in assessment is that, because Wikipedia articles are “living” documents, the edits of trainees may not be reflected in the article at the time of assessment. However, nothing is ever lost on Wikipedia [40]! We recommend using the article history to extract the contributions made by the trainee and carefully and fairly assess her contributions, not only on edits that remain in the current article, but also edits that may have been deleted or modified [42]. This approach may take longer than assessing a traditional term paper, which is wholly the work of the learner; one solution is to have the learner also document her edits, and present this as part of the submitted assignment.

## Rule 6: Provide learners with informative examples

Once you have set the expectations for the assignment, it is important to make sure learners understand those expectations and how to meet them. To do so, we recommend a two-step process. First, provide examples of both good and bad contributions, and second, have learners use your evaluation criteria to evaluate current articles. Providing robust models that illustrate specific features (see **Rule 7**) is an important part of writing pedagogy [43], and this is a first step in the process.

A helpful resource for locating examples of good Wikipedia articles specific to computational biology is Wikipedia’s Release Version Tool [33]. The article quality grades in this list are generated based on appraisals using the Wikipedia Content Assessment Scale discussed in **Rule 4**. From here, it is possible to find articles that are already in a good state (or in a poor state). A guided exercise to help learners understand the evolution of an article might include finding a good article, or even an ISCB award-winning article [44], and dissecting its editing history. Such an activity can help learners visualize potential improvements to currently underdeveloped articles. A next step might be to have learners practice using your rubric or evaluation criteria to evaluate good and bad articles, either as they currently appear or as they did at some point in the past.

Additionally, it may be helpful to introduce learners to the Wikipedia “talk” pages, which are administrative pages associated with each article. Editors use these pages to discuss the content, edits, and needs of articles. For example, the talk page for the Margaret Oakley Dayhoff article referenced in **Rule 4** contains justifications for past edits as well as recommendations for improvement that would be appropriate for an article that is of interest not only to those in computational biology but also to those who follow biographies or women’s history. Although they are sometimes underused, it is helpful to highlight these resources to learners so they can understand the expectations of different kinds of articles and their editors.

The degree of depth required at this stage depends on your learners. Students with more advanced writing skills may be able to readily appraise the current state of a Wikipedia article, while other students may require additional practice with more frequent teacher- or mentor-directed feedback.

## Rule 7: Offer guidance on genre-specific writing for Wikipedia

Learning how to write in a new genre or with different conventions can be a daunting task. While your learners may have significant experience writing in an academic setting, it is important to note that a Wikipedia-based writing assignment has some unique aspects—intended audience, source selection, and discourse patterns, for example—which contrast with a traditional term paper. There is also an inherent difference in the approach to collaborative writing and the types and immediacy of feedback (see **Rule 8**). A learner’s awareness of the differences between writing styles relates to ISCB core competency N (see **Table 1**).

The “Five pillars” are Wikipedia’s most fundamental principles [45,26]; the second “pillar” covers writing in a Wikipedia-appropriate style. We recommend that your trainees begin by reading the Ten Simple Rules for Editing Wikipedia [26] and completing one of the online courses for new Wikipedia editors [46,47]. The Wikimedia Foundation has also provided guidance on why writing for Wikipedia requires a different skill set than writing a traditional term paper [42]. Sharing these resources with your learners at the start of their learning experience will help to underline the differences and commonalities when writing for Wikipedia.

One important feature that distinguishes Wikipedia articles from academic articles or term papers is that original research is strictly not allowed, and primary sources are rarely used. To the surprise of some learners and especially educators (usually more accustomed to writing for journal papers), this policy may extend to primary source articles appearing in peer-reviewed journals, which should still be cited with care. Information added to Wikipedia should be based on reliable and published secondary or tertiary sources. For scientific topics, these are usually review articles. For computational biology, in particular, this creates a dilemma due to the fast-moving pace of the field. However, waiting for secondary sources on a particular topic helps to establish its notability (without which, articles may be swiftly deleted). The absence of review articles may also identify writing opportunities for educators and for advanced learners.

The technical differences when writing for Wikipedia necessitate additional considerations and learner training activities. For instance, some Wikipedia editing features can only be accessed through Wikipedia’s markup language, Wikitext, which is different from traditional word-processing software. We recommend suggesting to learners that they learn to use Wikitext, in the same way they may learn other similar tools, such as LaTeX; note that such web-based computing skills relate to ISCB core competency I (see **Table 1**). Learners can take low-stakes opportunities to practice the technical requirements of Wikipedia editing by experimenting in the Wikipedia Sandbox [48].

## Rule 8: Encourage learners to recognize that feedback is a gift

Good feedback is a gift [49]. Wikipedia-based writing projects offer a rare opportunity for learners to receive authentic feedback on their writing from subject-matter experts who serve as Wikipedia editors. Unlike a traditional term paper, which often is reviewed only by an instructor and/or peers, a Wikipedia article is a “living document,” monitored by editors who strive to provide consistent, accurate, and appropriate domain knowledge to readers. In doing so, the editors offer a form of evaluative (rather than descriptive) feedback that learners may not be accustomed to receiving.

To equip learners with the capacity to accept and make effective use of this authentic feedback, we recommend four strategies: (a) engage learners in the selection of articles to be edited (see **Rule 4**), (b) create an iterative cycle of editing and feedback with multiple revisions, similar to the revision cycles for scientific journal articles, (c) help learners discern feedback that is useful from feedback that is not useful, and (d) create opportunities for learners to provide quality feedback via peer review activities. These strategies engage learners in types of teamwork that align with ISCB core competency O (see **Table 1**). Educators will benefit from the authentic feedback of other Wikipedia editors by partially “crowdsourcing” their assessment of learners’ knowledge, because obvious errors will generally be swiftly reverted and plagiarized text will be removed (usually automatically, by one of Wikipedia’s software “bots”).

While most feedback will be constructive, it is important to remember that occasional conflicts may arise despite good faith on both sides. Indicating to regular editors that articles are being modified as part of a class assignment by using Wikipedia’s “educational assignment template” [50] may encourage those editors “not to bite the newbies” [51]. In a previous set of Ten Simple Rules, Dashnow and colleagues asked “how would Darwin have handled a Wikipedia edit war?” [52]. While this was asked in jest, we recommend that you give some forethought as to how you will deal with potential conflicts involving your learners and other Wikipedia editors. Wikipedia has a comprehensive dispute resolution procedure [53]; we suggest becoming familiar with this resource before such conflicts arise.

It should be emphasized that embracing constructive feedback and resolving conflicts in an ethical and considered manner are important parts of learners’ continuing professional development and are useful skills for interacting with journal article reviewers and thesis examiners, as well as in many contexts outside of academia. These skills fall under ISCB core competency M (see **Table 1**).

## Rule 9: Connect learners with the wider Wikipedia community

Wikipedia is almost entirely community led. Instead of having learners edit in a bubble, encourage them to connect with the wider Wikipedia community, including across languages. Making these connections will demonstrate to learners that their writing will be seen globally and should encourage them to consider the impact of their work in a wider context. Indeed, it has been observed that once a learner’s edits become live on Wikipedia and indexed in search engine results, they begin to realize that “there is agency to sharing their scholarship with the world” [54]. Of course, if you are not already registered with Wikipedia, we recommend doing so and spending some time editing Wikipedia and connecting with the community. The Wikipedia School and University projects page collects information about Wikipedia class projects and is home to a community of Wiki-friendly educators [55].

We also recommend joining the Computational Biology task force of WikiProject Molecular Biology. This task force, previously known as WikiProject Computational Biology [30], is an international community of Wikipedia editors formed in 2007 to organize and improve the roughly 1,500 Wikipedia articles relating to all aspects of computational biology and bioinformatics.

We encourage educators and learners alike to remain engaged with Wikipedia after their project, since they have subject-specific expertise that is enormously valuable for Wikipedia. For learners, in particular, remaining engaged with Wikipedia can provide an additional channel to keep up to date with new developments in a topic area in which they are interested. Topic Pages are a collaborative initiative between *PLOS* journals and Wikipedia [56] for review-type articles on subjects that are not covered in Wikipedia. These articles are published simultaneously on Wikipedia and in a *PLOS* journal and would be an ideal follow-up activity, especially for advanced learners. These connections and longer-term engagement with the Wikipedia community, and the consideration of the wider impact of a learner’s work, align with ISCB core competencies L and P (see **Table 1**).

## Rule 10: Share outcomes with other educators

After your learning experience is complete, take some time to reflect on the activity and share the outcomes with other educators. There are three major outcomes to be considered: (i) the impact of the project on the learner and the scale of the contribution to the public’s knowledge of computational biology, (ii) the design of the learning experience and how it can be improved, and (iii) how to share what you have learned to aid future educators.

The principal focus of your reflection should be on what learners have gained from completing a Wikipedia-based assignment. As discussed in **Rule 3**, it is helpful to consider how learners would benefit from such an assignment; this benefit may be measured. In addition to the specific learning outcomes of your course (e.g., the improved depth of knowledge about bioinformatics algorithms in a technical course), consider the growth of learners in general and transferable skills attained. The ISCB core competencies presented in **Table 1** may be useful for measuring these, especially when paired with a hierarchical model for classifying learning objectives (such as Bloom’s taxonomy [24,57]).

After completing their assignment, each trainee will have made some concrete contributions to the public’s knowledge of computational biology [3]. Wikipedia’s article quality ratings [34] may help quantify the improvements. Due to the nature of Wikipedia, the learner’s edits may last for years or may be changed by tomorrow. A high turnover of edits may be expected when writing about a fast-paced field such as bioinformatics; however, it is worth attempting to define the qualities of lasting edits and promoting these in future learning experiences, including future iterations of the course.

This previous point may guide reflection on the design of the learning experience. Inevitably, some aspects of the project will have been more successful than others, and we encourage an iterative approach to refining your learning experience. For instance, was there a significant variation in the quality of edits made by different learners, which might be remedied by a group-based element in future assessments? This iterative refinement of the project will be easier if the details of the entire process have been documented. Here, identifying and improving a single aspect of the project will be more successful than modifying many features at once.

Documenting and sharing your experiences and course materials with other educators demonstrates a commitment to Wikipedia’s ethos of openness and may encourage other educators to implement similar projects, continuing and expanding the cycle of knowledge transfer. This documentation may range from an informal write-up on Wikipedia, through resource sharing via the open Zenodo repository, to a short report submitted to a journal such as the *ISCB Community Journal* or presentation at the annual meeting of the ISCB Education COSI [58]. A deeper evaluation of your learning experience may be submitted as an educational review; we suggest involving your trainees as coauthors: the review by Chiang and colleagues is an excellent example of this [28]. We also recommend that trainees submit improved articles to the ISCB Wikipedia Competition [4,5]; as well as recognition from an international scholarly society, there is an additional financial incentive in the form of awards presented (at the Intelligent Systems for Molecular Biology conference) to the editors who have made the best improvements to their chosen article.

## Conclusion

There is an emerging consensus that editing Wikipedia articles as part of a class curriculum has lasting benefits far beyond the classroom. The scientific academic community remains wary of its relationship with Wikipedia [59], yet Wikipedia continues to be a first reference for many learners searching for information on unfamiliar topics. Both academia and Wikipedia would benefit from a stronger relationship and we believe that, for academics, improving Wikipedia articles represents not only an educational opportunity but a professional responsibility [29,60].

The increasing pace of the field of computational biology means that the many important Wikipedia articles are outdated or incomplete; as of July 2017, 80% of relevant articles had a Wikipedia quality rating of “start” class (articles that are developing but are essentially incomplete) or lower [30]; this state has persisted through the time of writing this article. We hope educators in computational biology will adopt the simple rules set out above to simultaneously enhance the learning of their students and improve a vital public resource for their profession. We believe that the replacement of traditional class projects with Wikipedia-based learning experiences is something to be embraced, and marks “[t]he end of throwaway assignments and the beginning of real-world impact for student editors” [10].
